# Strawberry Yield Improvement by Hydrogen-Based Irrigation Is Functionally Linked to Altered Rhizosphere Microbial Communities

**DOI:** 10.3390/plants13131723

**Published:** 2024-06-21

**Authors:** Longna Li, Huize Huang, Zhiwei Jin, Ke Jiang, Yan Zeng, Didier Pathier, Xu Cheng, Wenbiao Shen

**Affiliations:** 1 Laboratory Center of Life Sciences, College of Life Sciences, Nanjing Agricultural University, Nanjing 210095, China; lln2013034@njau.edu.cn (L.L.); 2023116075@stu.njau.edu.cn (H.H.); 2022816116@stu.njau.edu.cn (Z.J.); 2020816131@stu.njau.edu.cn (K.J.); 2 Air Liquide (China) R&D Co., Ltd., Shanghai 201108, China; yan.zeng@airliquide.com (Y.Z.); didier.pathier@airliquide.com (D.P.); steven.cheng@airliquide.com (X.C.)

**Keywords:** strawberry, yield, rhizophere microbiome, microbial functional gene, plant nutrient uptake

## Abstract

Molecular hydrogen (H_2_) is crucial for agricultural microbial systems. However, the mechanisms underlying its influence on crop yields is yet to be fully elucidated. This study observed that H_2_-based irrigation significantly increased strawberry (*Fragaria* × *ananassa* Duch.) yield with/without nutrient fertilization. The reduction in soil available nitrogen (N), phosphorus (P), potassium (K), and organic matter was consistent with the increased expression levels of N/P/K-absorption-related genes in root tissues at the fruiting stage. Metagenomics profiling showed the alterations in rhizosphere microbial community composition achieved by H_2_, particularly under the conditions without fertilizers. These included the enrichment of plant-growth-promoting rhizobacteria, such as *Burkholderia*, *Pseudomonas*, and *Cupriavidus* genera. Rhizobacteria with the capability to oxidize H_2_ (group 2a [NiFe] hydrogenase) were also enriched. Consistently, genes related to soil carbon (C) fixation (i.e., *rbcL*, *porD*, *frdAB*, etc.), dissimilar nitrate reduction (i.e., *napAB* and *nrfAH*), and P solublization, mineralization, and transportation (i.e., *ppx-gppA*, *appA*, and *ugpABCE*) exhibited higher abundance. Contrary tendencies were observed in the soil C degradation and N denitrification genes. Together, these results clearly indicate that microbe-mediated soil C, N, and P cycles might be functionally altered by H_2_, thus increasing plant nutrient uptake capacity and horticultural crop yield.

## 1. Introduction

Microorganisms, algae, and plants have capabilities to synthesize or metabolize molecular hydrogen (H_2_) [1,2]. Among these, microbial H_2_ cycling in agricultural soils is beneficial for plant growth [1]. For example, H_2_ production/leakage during N_2_-fixation by rhizobia stimulated the proliferation of H_2_-oxidizing bacteria (HOB), carbon (C) fixation, and soil enzyme activities in rhizospheric soil [3,4,5,6]. These findings were used to partially explain legume-associated benefits in crop rotation and plant growth promotion [3,5,6]. Further studies about rhizosphere bacteria and the surrounding soil revealed that numerous taxonomic groups, with Actinobacteria, Proteobacteria, Chloroflexi, and Acidobacteria dominating, harbor specific sequence-encoding high-affinity group 1h [NiFe]-hydrogenases, therefore scavenging a trace concentration of H_2_ [7,8,9]. Some Proteobacteria, including *Bradyrhizobium japonicum* and *Cupriavidus necator*, can use their low-affinity group 1d and 3d [NiFe]-hydrogenases to survive and grow on high levels of H_2_ [10]. Surprisingly, various taxa can grow efficiently on a wide range of concentrations of H_2_ using group 2a [NiFe]-hydrogenases [11,12]. Ample evidence further revealed that H_2_ exposure could influence soil biogeochemical processes and microbial communities [13].

Although the synthetic pathway of H_2_ in higher plants is not elucidated, several lines of evidence clearly discovered increased H_2_ production upon abiotic stress, such as drought [14], salinity [15], cold [16], and herbicide [17], as well as disease attack [18]. The involvement in plant responses was mostly validated when H_2_ was supplied with H_2_-enriched solution or its fumigation [14,15,16,17,18,19]. The physiological function of H_2_ in plant responses against salinity [20] and drought [21] was further confirmed using transgenic plants overexpressing hydrogenase 1 gene (*CrHYD1*) from *Chlamydomonas reinhardtii*. This genetic approach further confirmed that plant-based H_2_ can improve nitrogen uptake in Arabidopsis, particularly under low N supply conditions by targeting nitrate reductase, thus increasing seed size and yield [22].

Strawberry (*Fragaria* × *ananassa* Duch.) is cultivated globally and has a high commercial value. To cope with environmental and health concerns caused by excessive chemical fertilizer and pesticide application, seeking a more sustainable and low-carbon cultivation approach becomes an important issue. Compared to the above positive results obtained in the environmentally controlled laboratory conditions, the application of H_2_ might have potential in agricultural benefits. However, the H_2_ supplementation in field trials is a challenge for both researchers and farmers [23]. H_2_ gas infusion was usually used for investigation of the H_2_ effect on soil microbes, and the H_2_-pretreated soils were used for planting [3,5,6]. Wang et al. [24] had buried pipes in soils for field H_2_ infusion (1% H_2_). Therefore, this method for H_2_ supplementation is unpractical in agricultural production. Besides, H_2_ is used in a high concentration, where the inflammability of H_2_ has to be considered. Due to the sensitivity of plants to H_2_ (0.0004~0.0016‰), a high concentration of H_2_ infusion might have a negative effect on plant growth [19]. Comparatively, H_2_ dissolved water is the better method for its easy operation and high safety (H_2_ concentration < 0.0016‰). Our previous field results show that the preharvest irrigation of hydrogen nanobubble water (HNW) can efficiently alleviate the negative effects of fertilizers on strawberry fruit aroma, thus improving consumer preferences [25].

Based on the above results, by using a metagenomic approach, this study aims to further investigate the influence of H_2_ supply on rhizosphere microbial communities and their possible physiological importance in both yield and quality traits of strawberries in the field conditions with or without nutrient fertilization.

## 2. Results

### 2.1. Strawberry Yield Promotion Achieved by H_2_-Based Irrigation

**Figure 1:**
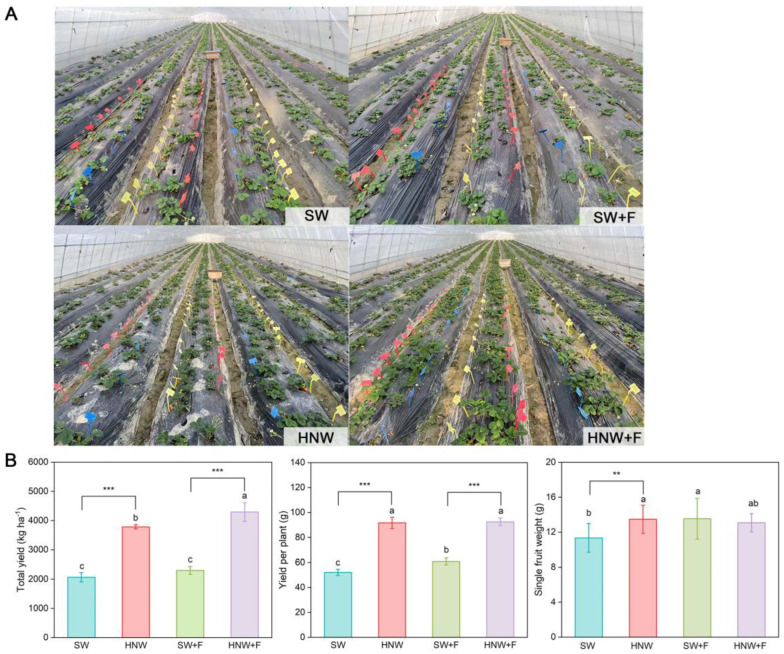
Hydrogen-based irrigation positively improves strawberry yield. Photographs of strawberry fields were taken on 28 December 2020 (**A**). Three replicates of 9 m^2^ plot were set up, and all soils in 12 plots were mixed and refilled from a 1.5 m depth in all plots. Corresponding total yield, yield per plant, and single fruit weight of strawberry (**B**) were then determined and calculated from 18 December 2020 to 6 April 2021. SW: surface water; HNW: hydrogen nanobubble water; SW + F: surface water plus fertilizers; HNW + F, hydrogen nanobubble water plus fertilizers. Data presented as mean ± SD (n = 3). Different letters indicate significant differences (*p* < 0.05) according to Tukey’s test. ** and *** indicate *p* < 0.01 and 0.001, respectively (*t*-test).

Field experimental data from two growing seasons showed that H_2_-based irrigation (HNW) exhibited positive effects on strawberry growth and yield, regardless of nutrient fertilization (Figure 1A, Supplementary Figure S1 and Table S1). For example, HNW irrigation caused significant increases in total yield (87.7 ± 17.8% and 82.9 ± 9.1%) and yield per plant (76.10 ± 0.58% and 52.04 ± 1.85%) of strawberry in the absence and presence of fertilizers, calculated from 18 December 2020 to 6 April 2021 (Figure 1B). Additionally, the single fruit weight was significantly increased by H_2_-based irrigation only under fertilizer-free condition. However, no significant difference was observed between the SW + F and HNW + F groups.

### 2.2. Altered Rhizosphere Microbial Community Structure by H_2_

**Figure 2:**
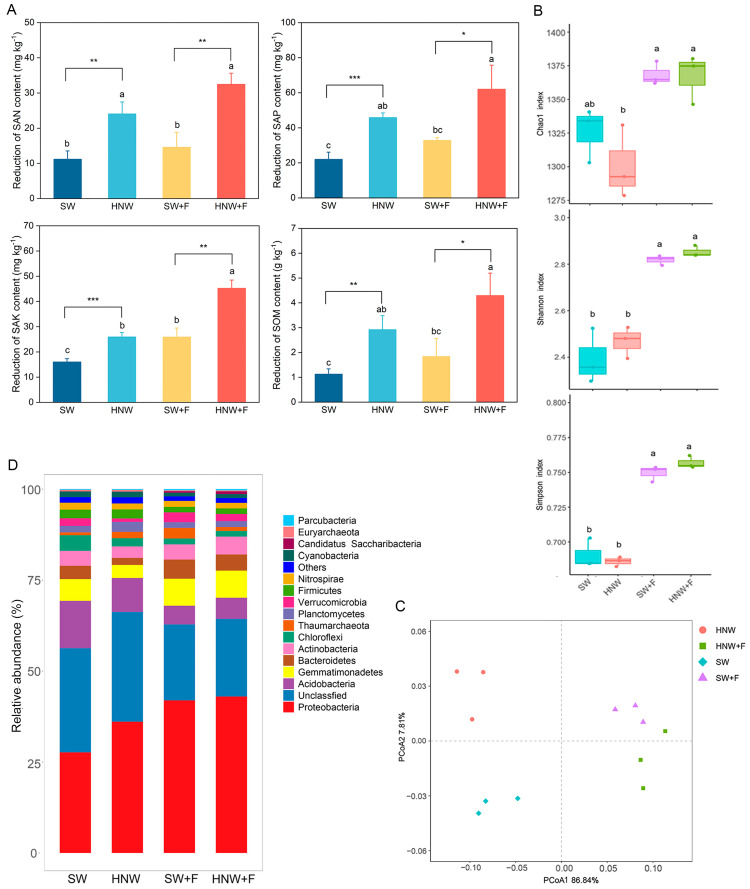
Changes in soil nutrients, diversity and composition of rhizosphere microbial communities in response to hydrogen-based irrigation. Reductions of soil available nitrogen (SAN), soil available phosphorus (SAP), soil available potassium (SAK), and soil organic matter (SOM) contents were calculated at the fruiting stage (10 November 2020) compared to that before planting (24 August 2020), respectively (**A**). Data presented as mean ± SD (n = 3). Boxplot showing Chao1, Shannon, and Simpson indexes of microbial communities (**B**). Principal coordinates analysis (PCoA) of the bacterial community composition based on Bray–Curtis dissimilarity at the genus level (**C**). Bacterial community composition on phylum level (**D**). Different letters indicate significant differences (*p* < 0.05) according to Tukey’s test. *, ** and *** indicate *p* < 0.05, 0.01, and 0.001, respectively (*t*-test).

In our field trial experiments, soil nutrient levels were arranged to be comparable among four treatments prior to transplanting (Supplementary Figure S2). However, at the fruiting stage, significant decreases in soil available nitrogen (SAN), soil available phosphorus (SAP), soil available potassium (SAK), and soil organic matter (SOM) contents were clearly observed in the HNW and HNW + F groups compared to SW and SW + F, respectively (Figure 2A). The effects of H_2_-based irrigation on rhizospheric microbial communities were subsequently investigated. We discovered that HNW irrigation did not significantly influence alpha diversity (i.e., Chao1, Shannon, and Simpson indexes) of the microbial communities, regardless of whether the fertilizer was added or not (Figure 2B). However, among four treatment groups, distinct separation of the genera of microbial communities was clearly observed based on the principal coordinate analysis (PCoA) with the first two principal coordinates, which, respectively, explained 86.84% and 7.81% of the variance (Figure 2C). This significant difference among these four treatments was supported by analysis of similarities (ANOSIM; R = 0.963, *p* < 0.0001; Supplementary Figure S3).

At the phylum level, the bacterial communities were dominated by Proteobacteria (37.17%), Acidobacteria (8.33%), Gemmatimonadetes (6.12%), Actinobacteria (4.04%), Bacteroidetes (3.82%), Chloroflexi (2.42%), Firmicutes (1.98%), Planctomycetes (1.93%), Verrucomicrobia (1.92%), Nitrospira (1.67%), Thaumarchaeota (1.60%), and Cyanobacteria (1.3%) (average abundances > 1%; Figure 2D, Supplementary Table S2). In the absence of fertilizer, significant increases achieved by H_2_-based irrigation was only observed in the abundance of Proteobacteria and Planctomycetes phyla (increased by 30.54% and 52.78%; *p* < 0.05) when compared to the SW group. Above enrichments were notably driven by *Burkholderia*, *Cupriavidus*, and *Pseudomonas* genera, exhibiting notable increases of 43.28%, 109.52%, and 27.45% (*p* < 0.05; Supplementary Table S3). Comparatively, among the dominant phyla, no taxa showed such significant differences between HNW + F and SW + F, except for Thaumarchaeota, showing a decreasing pattern (Supplementary Table S2). We subsequently observed that the genera *Sphingomonas*, *Novosphingobium*, and *Sphingobium* of Proteobacteria in the HNW + F group, as well as *Mycobacterium* of Actinobacteria, were more abundant (increased by 71.96%, 102.29%, 50.00%, and 11.11%, respectively; *p* < 0.05 or 0.01), compared to SW + F treatment (Supplementary Table S3).

The subsequent analysis revealed that, compared to the SW group, Proteobacteria encoding group 2a [NiFe]-hydrogenases and group C1 and C2 [FeFe]-hydrogenases exhibited significant enrichment in the HNW treatment (Supplementary Figure S4 and Table S4). In the presence of fertilizers, Chloroflexi encoding group 2b [NiFe]-hydrogenases, and other minority bacterium encoding group 1f [NiFe]-hydrogenases, were enriched by H_2_-based irrigation.

### 2.3. Function Potentials of Rhizosphere Microbial Community in Response to H_2_

**Figure 3:**
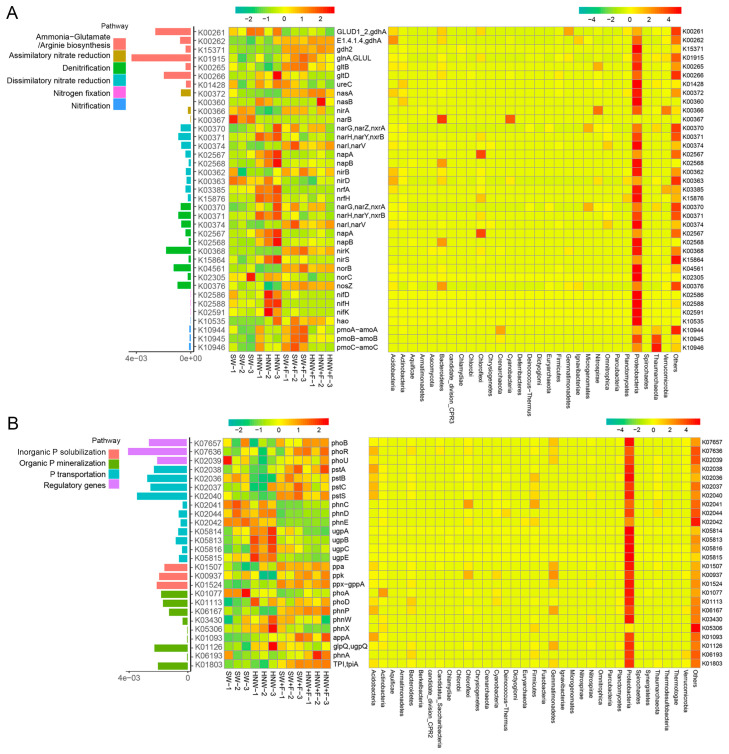
Relative abundances of genes involved in soil carbon fixation (**A**) and degradation (**B**) in response to hydrogen-based irrigation. Bars indicate the total relative abundances across all treatments. The middle heatmap represents the relative abundances in each sample, and the right represents the relative abundances of genes in each dominant phylum.

As shown in Figure 3A and Supplementary Table S5, subsequent results showed that, under our experimental conditions, *ACAT*, *tktA*, and *coxL* were the most abundant genes involved in C fixation, where the relative abundances of many genes were significantly higher in the HNW group than those in SW. These genes in Proteobacteria, for example, include *PRK* (2.25-fold) and *rbcL* (1.86-fold) related to the Calvin cycle, *porA* (1.53-fold) and *frdA* (3.69-fold) related to the rTCA cycle, *coxL* (1.60-fold) related to the Wood–Ljungdah pathway, *ACAT* (1.26-fold) related to the HP/HB cycle, etc. (*p* < 0.05 or 0.01). However, there were no such significant differences between the HNW + F and SW + F groups.

Conversely, for C degradation, most genes exhibited lower abundances in the HNW group than those in SW, including *amyA* (0.26-fold) and *malZ* (0.49-fold) involved in starch degradation, *bglX* (0.64-fold), and *bglB* (0.68-fold) associated with cellulose degradation, *abfA* (0.47-fold), *lacC* (0.54-fold) and *bgaB* (0.36-fold) related to hemicellulose degradation, etc. (Figure 3B and Supplementary Table S5; *p* < 0.05 or 0.01). By contrast, only four genes were altered significantly in response to HNW under the fertilized conditions, including *katG* (1.27-fold), *treZ* (0.89-fold), *SGA1* (0.87-fold), and *glcD* (0.87-fold). Notably, the genes involved in C degradation were more abundant in Proteobacteria, Acidobacteria, and Bacteroidetes compared to other phyla.

**Figure 4:**
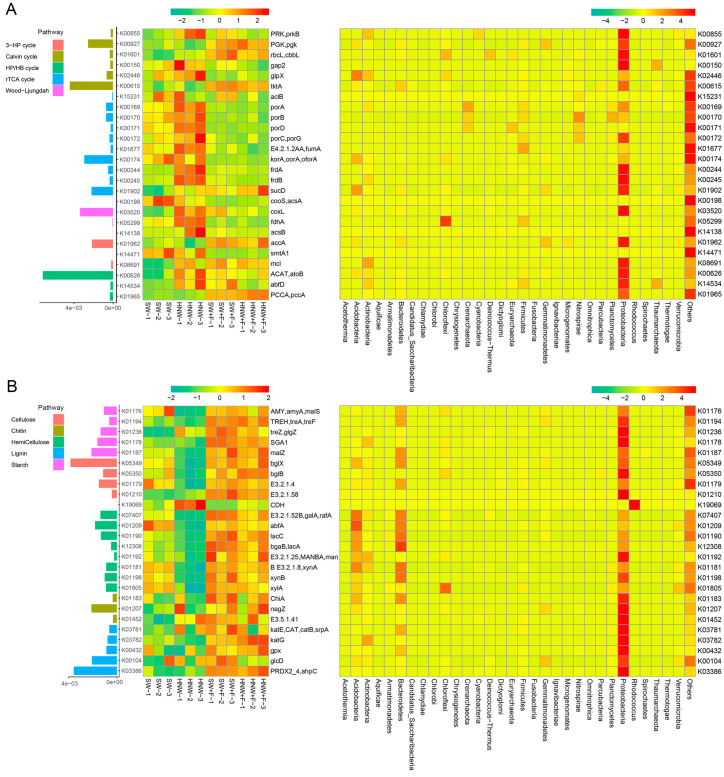
Relative abundances of genes involved in soil nitrogen (**A**) and phosphorus cycling (**B**) in response to hydrogen-based irrigation. Bars indicate the total relative abundances across all treatments, and the middle heatmap represents the relative abundances in each sample, and the right represents the relative abundances of genes in each dominant phylum.

In the absence of fertilizers, *napA* (2.63-fold), *napB* (3.07-fold), *nrfA* (2.01-fold), and *nrfH* (2.81-fold) for dissimilatory nitrate reduction to ammonium (DNRA) displayed higher abundances in the HNW group compared to SW in Proteobacteria, Chloroflexi, and Bacteroidetes (Figure 4A and Supplementary Table S5, *p* < 0.05 or 0.01). In contrast, *nirA* (0.30-fold) and *narB* (0.02-fold) for assimilatory nitrate reduction to ammonium (ANRA), *nirK* (0.80-fold), and *norB* (0.67-fold) for denitrification were less abundant in the HNW group. By contrast, fewer genes exhibited significant differences in the abundance when fertilizers were, respectively, added. For instance, only the relative abundance of *narB* (2.46-fold) for ANRA was higher, while *pmoA*/*amoA* (0.37-fold), *pmoB*/*amoB* (0.55-fold), and *pmoC*/*amoC* (0.38-fold) related to nitrification was lower in the HNW + F group, compared to those in SW + F treatment (*p* < 0.05 or 0.01).

For P cycling, *ugpA* (1.33-fold), *ugpB* (1.89-fold), *ugpC* (2.17-fold), and *ugpE* (1.36-fold) for P transportation, *ppx-gppA* (1.16-fold) for inorganic P solubilztion, and *phoD* (1.22-fold) and *appA* (7.72-fold) for organic P mineralization displayed more abundant in the HNW alone group compared with its control (SW) in Proteobacteria (Figure 4B and Supplementary Table S5; *p* < 0.05 or 0.01). In the presence of fertilizers, *phnC* (1.51-fold), *phoA* (1.44-fold), and *phnA* (2.48-fold) involved in P transportation and mineralization were enriched by H_2_-based irrigation (*p* < 0.05 or 0.01).

### 2.4. H_2_ Response Is Functionally Linked to Rhizosphere Microbial Communities

**Figure 5:**
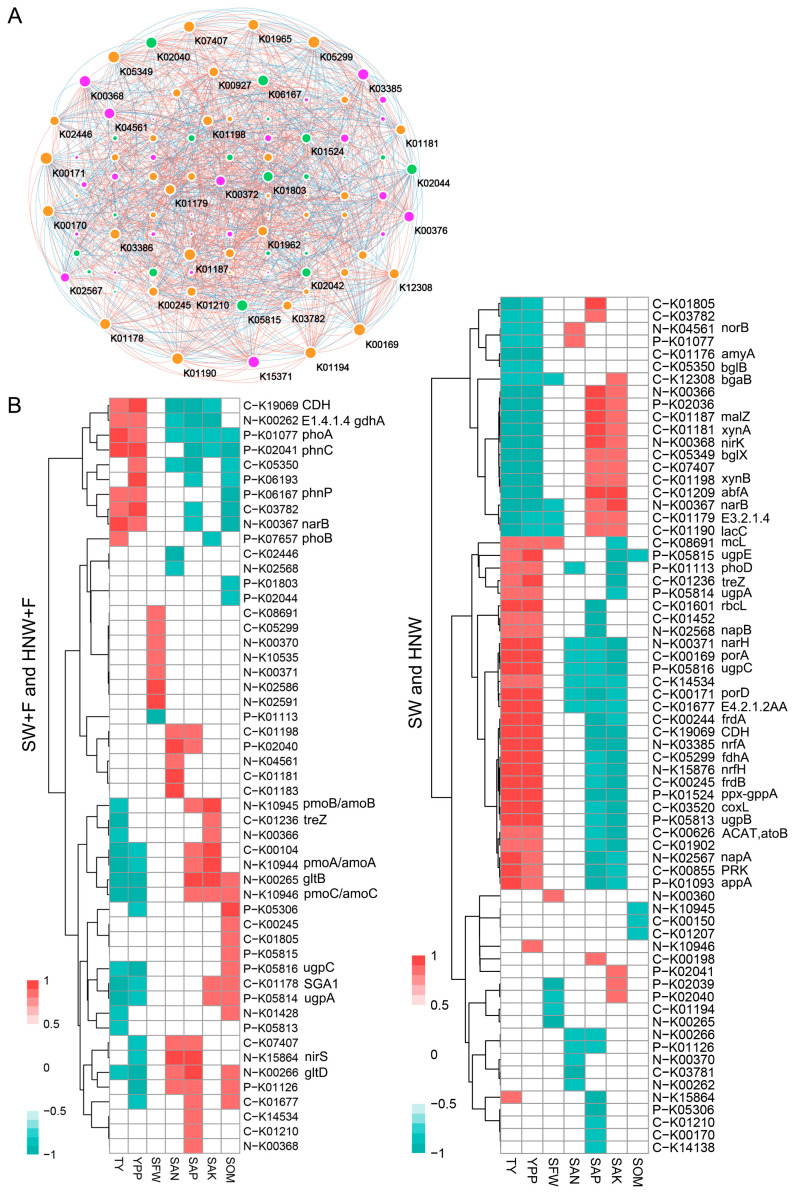
Correlations between key genes related to carbon, nitrogen, and phosphorus cycling in networks and strawberry yield in response to hydrogen-based irrigation. Yellow, green, and pink nodes represent genes involved in C, N, and P cycling, respectively (**A**). The size of each node is proportional to the number of connections. Red and blue lines, respectively, represent positive and negative linear relationships (|R| > 0.8, and *p* < 0.01). Pearson correlation coefficients between key genes in networks and yield or soil properties at the fruiting stage (10 November 2020; |R| > 0.8, and *p* < 0.01; (**B**)). SAK: soil available potassium content; SAN: soil available nitrogen content; SOM: soil organic matter content; SAP: soil available phosphorus content; SFW: single fruit weight; TY: total yield; YPP: yield per plant.

The network analysis showed a co-occurrence pattern among the genes related to C, N and P cycling. As shown in Figure 5A and Supplementary Table S6, this network consists of 97 nodes and 1087 edges. Notably, *porA* (K00169), *rbcL* (K01601), *nrfA* (K03385), *napA* (K02567), and *ugpE* (K05815), identified with the higher abundances in HNW compared to the SW group (Figure 3 and Figure 4), as well as *katG* (K03782) and *phnC* (K02041) exhibiting higher abundances in HNW + F compared to the SW + F group, also had strong and significant correlations with other genes (|R| > 0.8, *p* < 0.01).

In addition, there were more key genes positively related to strawberry yield (TY, YPP, and SFW) in the networks for the comparison between the SW and HNW groups, as well as that of the SW + F and HNW + F groups (Figure 5B and Supplementary Table S7; |R| > 0.8, *p* < 0.01). For example, genes related to C fixation (*frdAB*, *rbcL*, *porAD*), DNRA (*napAB* and *nrfAH*), and P solubilization, mineralization and transportation (*ppx-gppA*, *appA*, and *ugpABCE*) were positively associated with strawberry yield (TY and YPP) in the SW and HNW network. In the network of SW + F and HNW + F groups, for example, *phoA* and *phnC* for P cycling showed a positive correlation with strawberry yield (TY and YPP). On the other side, some key genes, including *bgaB*, *malZ*, *nirK*, *lacC*, etc., were positively associated with soil properties (particularly SAP and SAK) in the SW and HNW network, whereas a positive correlation was observed between key genes, including *gltB*, *pmoABC*/*amoABC*, *ugpAC*, etc., and soil properties (including SAP, SAK, or SOM) in the SW + F and HNW + F network.

**Figure 6:**
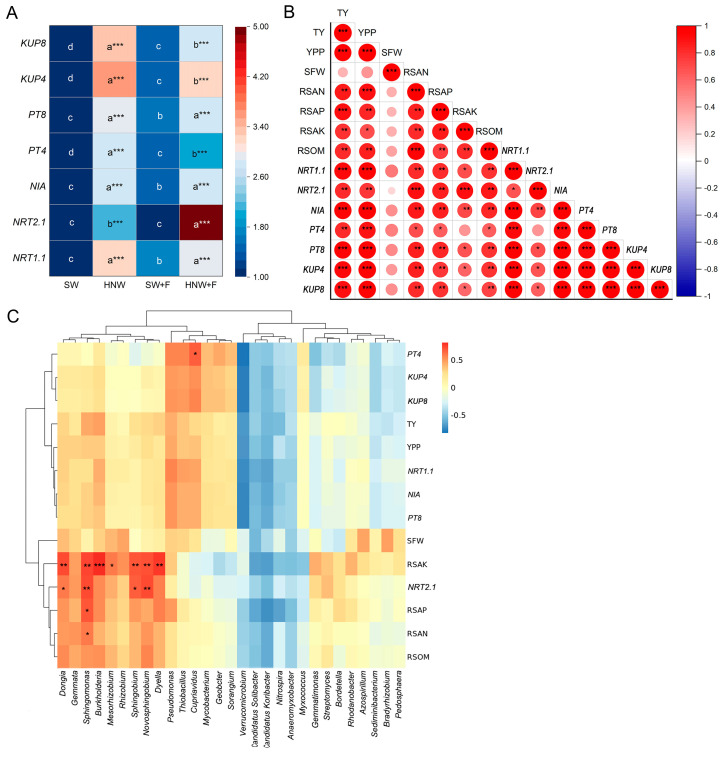
Correlation analysis underlying hydrogen-based irrigation improvement in strawberry yield was conducted by targeting the genes related to carbon, nitrogen, and phosphorus uptake in strawberries and rhizosphere microbes. Transcriptional profiles of genes related to N, P, and K absorption in strawberry roots at the fruiting stage (**A**), including nitrate transporter genes *NRT1.1* and *NRT2.1*, nitrate reductase gene (*NIA*), phosphate transporter genes *PT4* and *PT8*, and K transporter genes *KUP4* and *KUP8*. Pearson correlation analysis among strawberry yield, soil nutrients reduction, and related gene expression in strawberry (**B**). Pearson correlation analysis between rhizosphere microbial abundances and strawberry yield, or soil nutrients reduction, or related strawberry genes expression (**C**). Different letters denote significant differences (*p* < 0.05) according to Tukey’s test. *, **, and *** indicate *p* < 0.05, 0.01, and 0.001, respectively. RSAK: reduction of soil available potassium content; RSAN: reduction of soil available nitrogen content; RSOM: reduction of soil organic matter content; RSAP: reduction of soil available phosphorus content; SFW: single fruit weight; TY: total yield; YPP: yield per plant.

Significant increases in the transcriptional profiles of genes related to N (*NRT1.1*, *NRT2.1*, and *NIA*), P (*PT4* and *PT8*), and K (*KUP4* and *KUP8*) absorption in strawberry roots were observed at the fruiting stage after H_2_-based irrigation, regardless of whether the fertilizer was added (Figure 6A), which were consistent with the reductions in SAN, SAP, and SAK contents in soil (Figure 2A). Subsequent correlation analysis revealed strong, positive correlations among yield (TY and YPP) and the reductions in the SAN, SAP, SAK, and SOM contents, as well as transcripts for genes involving in N, P, and K absorption in strawberry roots, respectively (Figure 6B). Significant positive correlations were also observed among enriched microbial genera and soil nutrient reduction and transcripts of genes related to strawberry N, P, and K absorption (Figure 6C). For instance, *Burkholderia* showed a high correlation coefficient with RSAK (R = 0.83, *p* < 0.001), and *Novosphingobium*, *Sphingomonas*, and *Sphingobium* genera were significantly correlated with RSAK and *NRT2.1* expression level (R > 0.7, *p* < 0.05 or 0.01). *Sphingomonas* also exhibited strong correlations with RSAN (R = 0.60, *p* < 0.05) and RSAP (R = 0.70, *p* < 0.05), and *Cupriavidus* exhibited a positive correlation with *PT4* expression level (R > 0.7, *p* < 0.05) (Figure 6C). However, these genera did not show such strong correlations with strawberry yield (TY and YPP) (0.25 < R < 0.5, *p* > 0.05).

## 3. Discussion

### 3.1. H_2_-Based Irrigation Represents a Sustainable Approach for Strawberry Yield Improvement

Exploiting the benefits and corresponding mechanisms of H_2_-based irrigation improvement of both yield and quality traits represents a sustainable approach for low-carbon agricultural production [19]. This study integrated metagenomic and biochemical analysis to show that the altered rhizosphere microbial communities by H_2_-based irrigation might positively influence soil C, N, and P cycles, thus increasing plant nutrient uptake capacity and strawberry yield.

Similar to the positive results obtained from small-scale field studies of rice in Qingpu, Shanghai [26], and daylily in Suqian, Jiangsu Province [27], our field trials during 4-year growing seasons (2019–2023) confirmed that H_2_-based irrigation resulted in a remarkable increase in strawberry growth or yield under fertilization or non-fertilization conditions (Figure 1, Supplementary Figures S1 and S5, Table S1). Yield of H_2_-irrigated strawberry in 2022 and 2023 increased by 16.67~33.3% (planting area was extended to ~0.82 ha, with the conventional management using fertilizers, the yield was estimated by the growers). Therefore, our findings are significant for both basic and applied hydrogen biology.

Plant growth and yield are closely associated with soil nutrient dynamics [28]. Interestingly, our further results showed that H_2_-based irrigation in field trials led to a pronounced reduction in soil nutrients, regardless of whether fertilizers were present or not (Figure 2A). Accordingly, it is reasonable to deduce that above changes might be attributed to altered microbial nutrient metabolism and plant uptake.

### 3.2. Promotional Effect of H_2_ Might Be Linked with the Alterations in Rhizosphere Microbial Community Composition

In contrast to the data reported by Osborne et al. [29], several laboratories discovered that a high concentration of H_2_ infusion can influence soil microbial composition in environmentally controlled conditions [13,30,31]. For instance, elevated H_2_ directly decreased the abundance of Actinobacteria, while it increased Gammaproteobacteria [5]. Although H_2_-based irrigation failed to influence the diversity of rhizosphere microbial communities (assessed by Chao1, Shannon, and Simpson indexes; Figure 2B), our field trials also showed that microbial community composition was remarkably altered, especially in the absence of fertilizer (Figure 2C), suggesting a possible functional link between the alteration in microbial community composition and improved strawberry field achieved by H_2_.

Partially consistent with the results reported by Xu et al. [31], our subsequent experiment revealed that after H_2_-based irrigation, increases in group 2a and 2b [NiFe]-hydrogenases were observed in strawberry rhizospheres under both unfertilized and fertilized conditions (Supplementary Figure S4). These results clearly indicated that bacteria with the ability to sense and oxidize H_2_ might be activated in response to H_2_ supply and rapidly utilize this energy source for growth. Certainly, we also admitted that under more variable field conditions, H_2_ might be not the only driving factor influencing microbial survival, and other factors might include soil moisture, soil type, and plant species [24,31], which need to be considered in the future field trials using H_2_-based irrigation.

Changes in the composition of the rhizosphere microbial communities were observed at the phylum and genus levels in response to H_2_-based irrigation, especially under non-fertilization condition. For instance, the phyla Proteobacteria and Planctomycetes were enriched by H_2_-based irrigation, evidenced by significant increases in the relative abundances of several plant-growth promoting rhizobacteria (PGPRs), including the *Burkholderia*, *Pseudomonas*, and *Cupriavidus* genera (Figure 2C, Supplementary Tables S2 and S3). Previous evaluation showed that some species of above bacteria were functionally linked to plant growth and yield promotion by improving plant N, P, and K uptake [32,33]. One isolate of *Burkholderia* from soil adjacent to Hup^–^ soybean nodules could positively increase the root elongation of spring wheat seedlings and Arabidopsis biomass [3]. A recent study discovered positive correlations between the relative abundance of potential PGPRs and yield/biomass of plants (including maize, rice, and peanut; [34]). Accordingly, we further speculated that H_2_-based irrigation promotion of strawberry yield might be causally linked with the alterations in rhizosphere microbial community composition, especially facilitating the enrichment of PGPRs.

### 3.3. H_2_ Supply Displays the Positive Effects on Microbial-Mediated Soil C, N, and P Cycles

Since the changed structure of the rhizosphere microbial community can potentially lead to alterations in its function [35], C, N, and P cycling in the rhizosphere microbiome was subsequently analyzed. As anticipated, the metagenomic analysis revealed that the microbial C, N and P cycles differed significantly between H_2_ supply condition and the corresponding controls, especially in the unfertilized treatment. Previous studies showed that the capacity for C fixation, primarily through Calvin cycle (*rbcL*), was increased by H_2_ in wetland and upland soils [30]. Consistently, H_2_-based irrigation increased the relative abundance of genes involved in C fixation (e.g., Calvin cycle and reductive citrate cycle genes), while contrasting responses were observed in genes related to organic C degradation (e.g., cellulose- and hemicellulose-degradation genes), all of which were more pronounced upon the non-fertilization condition (Figure 3 and Supplementary Table S5). Previous studies showed that H_2_ stimulation of β/γ-Proteobacteria was accompanied by the increased net CO_2_ fixation [4]. Therefore, we speculated that an increase in the potential microbial C fixation and a reduction in microbe-mediated soil C loss might be achieved by H_2_-based irrigation.

For N cycling, controversial results of related works exist. For example, based on H_2_ infusion, one is that it did not impact the abundances of N cycle genes in the lawn kept free of vegetation [24], and another finding showed that the increased *napA* and *nirS* relative abundance and decreases in *norB* abundance in high (2% and 5%) H_2_-treated soils were present in the paddy wetland/meadow upland [30]. The data from the present study demonstrate that, under unfertilized conditions, the level of DNRA (i.e., *napAB* and *nrfAH*) were remarkably increased by H_2_ supply (Figure 4A and Supplementary Table S5), reflecting the possibility that the increased potential of soil NH_4_^+^-N derived from NO_3_^−^-N after H_2_-based irrigation, ultimately leading to strawberry growth and yield promotion due to the preference of strawberry for NH_4_^+^-N [36]. Since DNRA competes with denitrification for NO_3_^−^, genes (notably *nirK* and *norB*) for denitrification, the main N-loss process in the agricultural soil [37] were also less abundant by H_2_. By contrast, a previous result showed the increased N_2_O emissions and *nirK* abundance in soil adjacent to legume nodules and in HOB isolate-inoculated corn soil [38]. These discrepancies in nitrification and denitrification might be due to the microorganism responses to H_2_ supply in different plant species, and also in the various H_2_ supply conditions and concentrations used in each case.

Phosphate-solubilizing bacteria dominating in the agricultural soils can release recalcitrant phosphate, thus improving crop nutrient acquisition and crop productivity [39]. In the present study, certain phosphate-solubilizing microorganisms, such as *Burkholderia*, *Pseudomonas*, and *Sphingomonas*, were enriched by H_2_ supply (Figure 2D and Supplementary Table S3). The activated microbial P metabolism was also observed, which was evaluated by the increased abundance of genes responsible for P transportation (*ugpABCE*), inorganic P solubilization (*ppx-gppA*), and organic P mineralization (*phoD* and *appA*; Figure 4B and Supplementary Table S5). These results clearly implicated the improved microbial efficiency of utilizing and internalizing soil P, thus converting the unavailable P into available forms for the crop absorption [40].

Compared to conditions without fertilization, in the presence of fertilizers, the impact of H_2_-based irrigation on soil microbial communities was relatively weaker, which might be attributed to the addition of Bacillus. However, it also exhibited the similar tendencies in genes related to C degradation (*treZ*, *SGA1*, and *glcD*), nitrification (*pmoABC*/*amoABC*), and P mineralization and transportation (i.e., *phoA* and *phnC*) in response to H_2_. The results supported our previous hypothesis that H_2_ supply displayed the positive effects on microbial-mediated soil C and N loss reduction and P availability, regardless of whether fertilizers were added.

### 3.4. Plant Nutrient Uptake Capacity Might Be Activated by H_2_-Based Irrigation

Subsequent molecular evidence supported another possibility: the activation of plant nutrient uptake capacity. Strawberry *NRT1.1*, *NRT2.1*, *NIA*, and *NiR* are crucial genes for nitrogen uptake and assimilation [41,42]. Our previous results showed that H_2_ supplied by exogenous or genetic manipulation could increase nitrate uptake and seed size in Arabidopsis by regulating the dephosphorylation of nitrate reductase under controlled laboratory conditions [22] and positively improve quantitative traits of rice by up-regulating the expression of *NRT2.3* and *NiR* in field trials [26]. These findings may have implications for minimizing fertilizer use achieved by H_2_.

Meanwhile, three P transporter genes, *PT4*, *PT5*, and *PT8*, in strawberry have been identified and found to be highly induced by mycorrhiza, contributing to efficient P transport and acquisition [43]. In addition, fifteen K^+^ uptake transporter genes (*KUPs*) were previously identified in wild strawberry, with nine of them largely upregulated in response to K^+^ deficiency [44]. A further functional complementation experiment in bacterial mutant indicated that *FveKUP8* could utilize external K^+^ under the neutral proton environment.

Consistent with changes in soil nutrients with or without H_2_ supply (Figure 2A), the qPCR results clearly showed that transcripts of *NRT1.1*, *NRT2.1*, *NIA*, *PT4*, *PT8*, *KUP4*, and *KUP8* in strawberry roots at the fruiting stage were all significantly induced by H_2_, regardless of whether fertilizers were added (Figure 6A). These changes were strongly and positively correlated with the enhanced soil nutrient reduction and strawberry yield (Figure 6B).

Previous studies have documented ample evidence of PGPR stimulating the expression of plant nutrient uptake-related genes, thus facilitating nutrient absorption. For example, *Pseudomonas nitroreducens* strain IHB B 13561 could induce a high expression of *NRT2.1* in Arabidopsis and lettuce roots, thus enhancing their nitrate uptake and biomass [45]. *Pseudomonas* sp. strain P34-L, isolated from the wheat rhizosphere, increased phosphate and biomass accumulation by regulating *PT4* expression [46]. Accordingly, our study clearly suggested that the obvious enriched PGPR (i.e., *Burkholderia*, *Novosphingobium*, *Sphingomonas*, *Sphingobium*, and *Cupriavidus*) driven by H_2_ was positively correlated with expressions of N- and P-absorption-related genes in strawberries and soil nutrient reduction (Figure 6C). Certainly, the detailed mechanisms involved are not well known, and these need to be further addressed in the near future.

## 4. Materials and Methods

### 4.1. Plant Material and Experimental Design

The field trials were carried out in Qingpu Agriculture Base (31.2° N, 121.1° E), Shanghai, China, from 2019 to 2023. Based on a pilot trial (September 2019 to April 2020; Supplementary Figure S1), this field trial was continued in the following growing season (September 2020 to April 2021), and the temperature and precipitation information is shown in Supplementary Figure S6. Additionally, the trials on increasing field production under conventional conditions (with nutrient fertilization) achieved by H_2_-based irrigation were further validated from 2021 to 2023 (Supplementary Figure S5).

Strawberries (*Fragaria* × *ananassa* ‘Benihoppe’) were provided by the farm where the base was located and were planted in a greenhouse (80 m long × 6 m wide) on 5 September 2020, in two rows per ridge with a 40 cm row spacing and a 25 cm plant spacing. There were a total of four treatments, one treatment per greenhouse: (1) irrigation of surface water without fertilizer application (SW); (2) irrigation of HNW without fertilizer application (HNW); (3) irrigation of surface water with fertilizer application (SW + F); (4) irrigation of HNW with fertilizer application (HNW + F). Among these, there were three replicates of 9 m^2^ plot per treatment, and all soils in 12 plots were fully mixed and refilled from a 1.5 m depth in all plots. Accordingly, the initial nutrient properties of soils were at the same level (Supplementary Figure S4), having soil available nitrogen (SAN), soil available phosphorus (SAP), soil available potassium (SAK), and soil organic matter (SOM) contents of 155.0 ± 2.3 mg kg^−1^, 151.5 ± 1.5 mg kg^−1^, 160.6 ± 1.6 mg kg^−1^, and 22.0 ± 0.5 g kg^−1^, respectively.

As previously reported [25], each greenhouse received the same independent management for water and pesticides that followed typical local agricultural practices. The base fertilizer consisted of 15,625 kg ha^−1^ organic fertilizer (Lüyuan Organic Fertilizer, Shanghai Yuanjian Organic Fertilizer Factory, Shanghai, China), 312.5 kg ha^−1^ composite fertilizer (KALI NPK + Mg, 13-7-25, Shenzhen K + S Group Trading Co. Ltd., Shenzhen, China), and 62.5 kg ha^−1^ composite Bacillus (Polysea, Yuncheng Bei Hai Chemical Co., Ltd., Yuncheng, China), all of which were applied in two green houses before planting. After 40 days of cultivation, an additional 416.7 kg ha^−1^ of composite fertilizer was applied in two greenhouses.

The HNW (~300 nm nanobubbles, ~1.0 mg H_2_ L^−1^, and ~12 h residence time of H_2_) was obtained by a H_2_ nanobubble generator from Air Liquide (China) R&D Co., Ltd. [25]. Immediately, the fresh HNW was pumped into the greenhouse (10 t h^−1^ of flow rate). In this trial, 20 t per greenhouse of HNW was irrigated every day for the first four days after planting, and 10, 5, and 30 t per greenhouse of HNW were irrigated on the fifth, sixth, and 40th day, respectively, according to the growth of plants and the previous experimental experience. Meanwhile, the strawberries in another two greenhouses were irrigated with SW (with or without nutrient fertilization), and were set as corresponding controls.

### 4.2. Soil Sample Collection and Analysis of Soil Physicochemical Properties

Soil samples were collected on 19 August 2020 (before fertilization), 24 August 2020 (after fertilization and before planting), and 10 November 2020 (at the fruiting stage) to measure soil nutrient properties, and samples from the third collection were also used for a metagenomic analysis. The rhizosphere soils were obtained by brushing soil attached to the roots [47]. Soil samples were collected in triplicate, and each contained the mixed soil from five sites. After natural air-drying, grinding, and sieving, the samples were used for the determination of soil nutrients. Besides, soil samples for metagenomic analysis were transported to the laboratory in liquid nitrogen and stored at −80 °C.

The SAN content was determined by the alkali solution diffusion method [48]. SAP and SAK were extracted with ammonium fluoride-hydrochloric acid and ammonium acetate solution (1 M, pH 7.0), respectively, and then were measured using Inductively Coupled Plasma Optical Emission Spectrometry (ICP-OES; Optima 8000, Perkin Elmer, Waltham, MA, USA; [49]). SOM was measured by the dichromate method, colorimetrically using a glucose standard, and calculated by multiplying a conversion factor of 1.724 [50]. All results were expressed as mg or g kg^−1^ dry soil. The reductions of SAN, SAP, SAK, and SOM were calculated at the fruiting stage (10 November 2020) compared to those before planting (24 August 2020), respectively.

### 4.3. Soil Metagenomic Sequencing and Analysis

Soil DNA was extracted following the manufacturer’s instruction for the PowerSoil DNA Isolation Kit (MoBio, Carlsbad, CA, USA). The concentration and integrity of the isolated DNA was estimated using a Qubit 3.0 Fluorometer (Thermo Fisher, Waltham, MA, USA) and 1% agarose gel electrophoresis, respectively.

DNA library (300–400 bp DNA) was constructed and sequenced on the DNBSEQ platform (MGI Tech, Shenzhen, China). Numbers of sequencing reads were showed in Supplementary Table S8. The raw data were quality-controlled using SOAPnuke (v.2.2.1) [51]. Afterwards, the clean data were assembled de novo using MEGAHIT (v.1.1.3) software [52]. Genes were predicted over contigs (>500 bp) using MetaGeneMark [53], and predicted genes with ≥95% identity and ≥90% coverage were clustered using CD-HIT to remove the redundant genes [54]. Salmon (v.1.3.0) software was used for quantification to obtain the standardized gene abundance [55]. The protein sequences of genes were aligned against the public database using DIAMOND blastp with an E value cutoff of 1 × 10^−5^ [56]. Moreover, MEGAN (version 5) was used for taxonomic annotations of each sample according to the National Center for Biotechnology Information (NCBI) taxonomy [57]. The species abundance in the sample was calculated by aggregating the abundance of gene annotated by the same species.

### 4.4. Strawberry Yield

Strawberry fruits in three replicates of 9 m^2^ plot were collected once per week during the harvesting season (from 18 December 2020 to 6 April 2021), and weight and quantity recorded. The total yield (TY), yield per plant (YPP), and single fruit weight (SFW) was then calculated.

### 4.5. Quantitative Real-Time PCR

RNA in root tissues was extracted according to the instruction of FastPure Plant Total RNA Isolation Kit (Vazyme, RC401), and its concentration and quality were estimated with a NanoDrop 2000 spectrophotometer (Thermo Fisher Scientific). Afterwards, cDNAs were synthesized using HiScript III RT SuperMix (+gDNA wiper; Vazyme (Nanjing, China), R323). qPCR was performed on a Mastercycler ep^®^ realplex real-time PCR system (Eppendorf, Hamburg, Germany) using TransStart Top Green qPCR SuperMix (Transgen (Beijing, China) AQ131). Relative expression levels of genes were derived by the 2^−ΔΔCT^ method [58] and normalized to reference genes (*Actin* and *18S rRNA*). The expression value was presented as a value relative to the control sample (SW treatment). Primers are shown in Supplementary Table S9.

### 4.6. Statistical Analysis

Values were presented as mean ± standard deviation (SD) from three independent experiments. Statistical analyses of yield, soil properties and gene expression levels among treatments, as well as the Pearson correlation coefficient from the above characters were calculated using SPSS 22.0 (SPSS Inc., Chicago, IL, USA). Meanwhile, R software (V 4.3.2) was used for metagenomic analysis. The alpha diversity (including Chao1, Shannon, and Simpson indexes) of soil microbial communities was quantified based on the taxon profile. Principal coordinate analysis (PCoA) and analysis of similarities (ANOSIM) was used to illustrate differences among the samples using Bray–Curtis dissimilarity at the genus level. The Pearson correlation coefficient was used to conduct network analysis of genes related to C-, N-, and P-cycling and to test the correlation significance among strawberry yield, soil properties, and above functional gene abundances. Prior to the ANOVA, the data were tested to ensure it was normally distributed (*p* > 0.05). One-way ANOVA followed by the Tukey’s test and *t*-test was used to evaluate differences between treatments.

## 5. Conclusions

**Figure 7:**
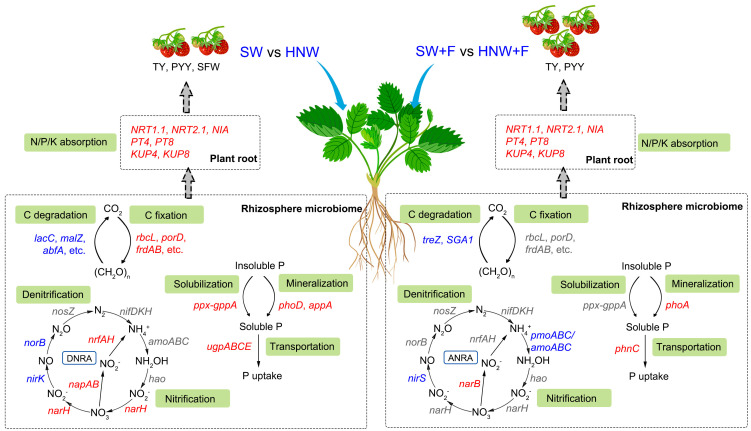
An overall schema illustrating the contribution of microbe-mediated soil carbon, nitrogen, and phosphorus cycles and plant nutrient uptake capacity in strawberry yield improvement by hydrogen-based irrigation. The colors of genes denote alterations in abundance or expression in rhizosphere microbial communities or strawberry roots achieved by hydrogen nanobubble water (HNW), compared to the corresponding control, respectively. Red indicates a significant increase; blue indicates a significant decrease; and gray indicates no significant difference.

As summarized in Figure 7, the integrated metagenomic and biochemical evidence presented in this study characterized a mechanism for H_2_-based irrigation function in strawberry yield improvement, possibly achieved by the alteration of rhizosphere microbial communities and the increased plant nutrient uptake capacity. The contribution of microbe-mediated soil C, N, and P cycles was elucidated as well. However, it should be noted that the microbial effects achieved by H_2_ might be influenced by plant species and environmental conditions. Since the possibility that the potential negative effects of H_2_ on soil ecosystems are not easily ruled out, the agricultural application of H_2_ requires the large-scale and long-term field trials. Overall, the results of this study will not only widen our basic knowledge of hydrogen biology but very likely also improve our understanding of low-carbon H_2_-based agriculture that may increase crop (including horticulture, etc.) yield in a sustainable manner.

## Data Availability

The metagenomic sequencing dataset are available in FigShare (DOI: 10.6084/m9.figshare.26064313).

## Supplementary Materials

The following supporting information can be downloaded at: https://www.mdpi.com/article/10.3390/plants13131723/s1, Figure S1: Hydrogen-based irrigation improves strawberry growth; Figure S2: Changes in soil available nitrogen (SAN; A), soil available phosphorus (SAP; B), soil available potassium (SAK; C), and soil organic matter (SOM; D) contents after HNW irrigation without/with fertilizers; Figure S3: Analysis of similarities (ANOSIM) based on Bray–Curtis dissimilarity at the genus level; Figure S4: Relative abundances of rhizospheric hydrogenase genes; Figure S5: Photographs of strawberry field and representative plants were taken on 14 January 2021 (A) and 14 February 2023 (B); Figure S6: The monthly maximum and minimum temperature and total precipitation during strawberry planting. Table S1: Strawberry yield in response to hydrogen-based irrigation; Table S2: Results of one-way analysis of variance (ANOVA) and *t*-test between treatments for Figure 2D; Table S3: Relative abundances (%) of dominant genera (Top 30) of bacterial community; Table S4: Results of *t*-test between treatments for Figure S4; Table S5: Results of *t*-test between treatments for Figure 3 and Figure 4; Table S6: Results of Pearson’s correlation among carbon, nitrogen, and phosphorus cycling genes for Figure 5A; Table S7: Results of Pearson’s correlation among genes related to carbon, nitrogen, and phosphorus cycling, yield and soil properties for Figure 5B; Table S8: Overview of soil metagenome sequencing and annotation results; Table S9: Primers used for qPCR.
