# Supplementary material for: Strawberry Yield Improvement by Hydrogen-Based Irrigation Is Functionally Linked to Altered Rhizosphere Microbial Communities
Source: Plants (Basel). 2024 Jun 21;13(13):1723. doi: 10.3390/plants13131723 (PMC11243525; doi:10.3390/plants13131723)
Supplement: Supplementary file 1 [file plants-13-01723-s001.zip › Plants-Supplementary Figures.pdf]

Supplementary Information

Strawberry Yield Improvement by Hydrogen-Based Irrigation Is Functionally Linked to Altered Rhizosphere Microbial Communities

Longna Li [1], Huize Huang [1], Zhiwei Jin [1], Ke Jiang [1], Yan Zeng [2], Didier Pathier [2], Xu Cheng [2] and Wenbiao Shen [1,*]

1 Laboratory Center of Life Sciences, College of Life Sciences, Nanjing Agricultural University, Nanjing 210095, China; lln2013034@njau.edu.cn (L.L.); 2023116075@stu.njau.edu.cn (H.H.); 2022816116@stu.njau.edu.cn (Z.J.); 2020816131@stu.njau.edu.cn (K.J.)

2 Air Liquide (China) R&D Co., Ltd., Shanghai 201108, China; yan.zeng@airliquide.com (Y.Z.); didier.pathier@airliquide.com (D.P.); steven.cheng@airliquide.com (X.C.)

* Correspondence: wbshenh@njau.edu.cn; Tel.: +86-25-84-399-032; Fax: +86-25-84-396-542

Table Legends

Table S1 Strawberry yield in response to hydrogen-based irrigation. The pilot trial was carried out from September 2019 to April 2020 (n = 1). The field trial was continued in the following growing season (September 2020 to April 2021), with arrangement of three plots per treatment. The data were expressed as mean ± SD (n = 3).

Table S2 Results of one-way analysis of variance (ANOVA) and *t*-test between treatments for Figure 2D. The data were expressed as mean ± SD (n = 3). Different letters denote significant differences ($P < 0.05$) according to Tukey's test. * indicates significant differences ($P < 0.05$) from the corresponding control (SW or SW+F), respectively (*t*-test).

Table S3 Relative abundances (%) of dominant genera (Top 30) of bacterial community. The data were expressed as mean ± SD from three replicates. Different letters denote significant differences ($P < 0.05$) according to Tukey's test. * and **

indicates significant differences (P < 0.05 and 0.01) from the corresponding control (SW or SW+F), respectively (t-test).

Table S4 Results of t-test between treatments for Supplementary Figure S4. *, **, or *** indicates significant differences at P < 0.05, 0.01, or 0.001 from the corresponding control (SW or SW+F), respectively.

Table S5 Results of t-test between treatments for Figures 3 and 4. *, **, or *** indicates significant differences at P < 0.05, 0.01, or 0.001 from the corresponding control (SW or SW+F), respectively.

Table S6 Results of Pearson's correlation among carbon, nitrogen, and phosphorus cycling genes for Figure 5A.

Table S7 Results of Pearson's correlation among genes related to carbon, nitrogen, and phosphorus cycling, yield and soil properties for Figure 5B.

Table S8 Overview of soil metagenome sequencing and annotation results.

Table S9 Primers used for qPCR.

Figure Legends

Figure S1 Hydrogen-based irrigation improves strawberry growth. Photographs of strawberry field and representative plants were taken on January 5, 2020.

Figure S2 Changes in soil available nitrogen (SAN; A), soil available phosphorus (SAP; B), soil available potassium (SAK; C), and soil organic matter (SOM; D) contents after HNW irrigation without/with fertilizers. SW, HNW, SW+F, and HNW+F are the rhizosphere soil samples corresponding to surface water or hydrogen nanobubble water

irrigation, without/with fertilizers. Data were expressed as mean ± SD from three replicates. Different letters denote significant differences (P < 0.05) according to Tukey's test.

Figure S3 Analysis of similarities (ANOSIM) based on Bray-Curtis dissimilarity at the genus level.

Figure S4 Relative abundances of rhizospheric hydrogenase genes. Bars indicate the total relative abundances of hydrogenase genes across all treatments. The middle heatmap represents the relative abundances in each sample, and the right represents the relative abundances in each dominant phylum.

Figure S5 Photographs of strawberry field and representative plants were taken on January 14, 2021 (A) and February 14, 2023 (B).

Figure S6 The monthly maximum and minimum temperature and total precipitation during strawberry planting.

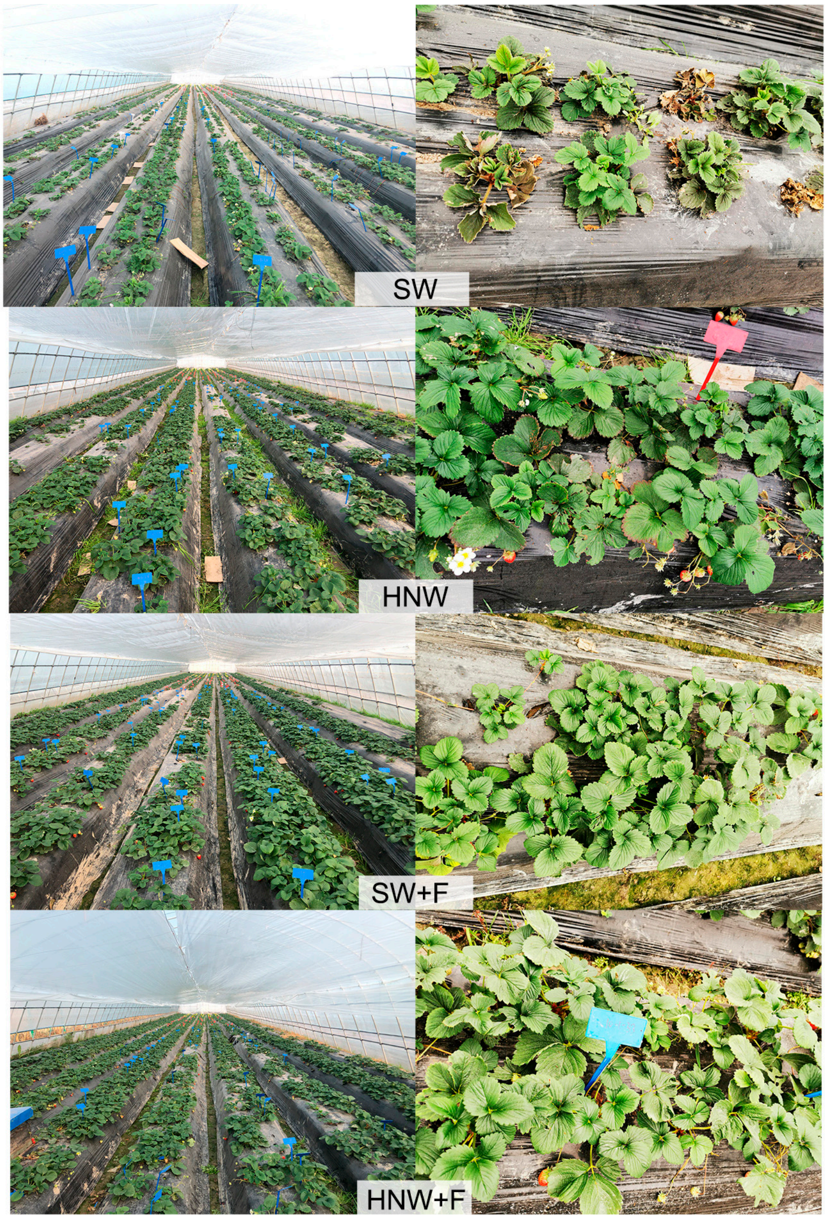

Figure S1 Hydrogen-based irrigation improves strawberry growth. Photographs of strawberry field and representative plants were taken on January 5, 2020.

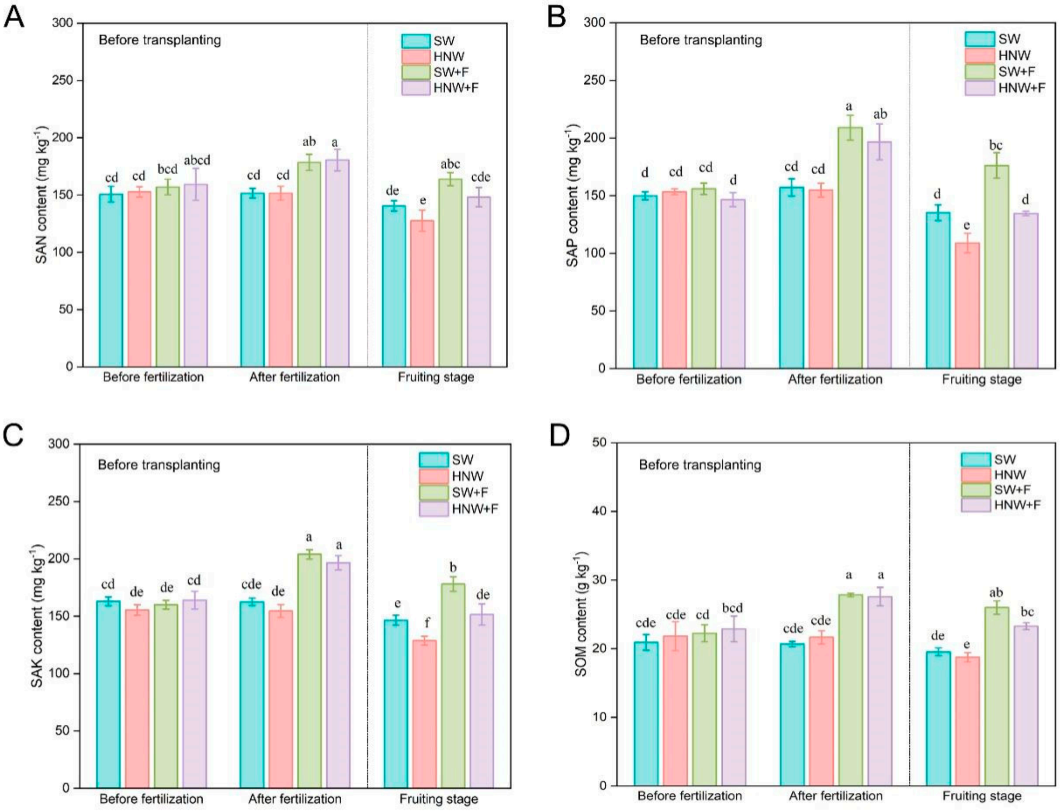

Figure S2 Changes in soil available nitrogen (SAN; A), soil available phosphorus (SAP; B), soil available potassium (SAK; C), and soil organic matter (SOM; D) contents after HNW irrigation without/with fertilizers. SW, HNW, SW+F, and HNW+F are the rhizosphere soil samples corresponding to surface water or hydrogen nanobubble water irrigation, without/with fertilizers. Data were expressed as mean ± SD from three replicates. Different letters denote significant differences (P < 0.05) according to Tukey's test.

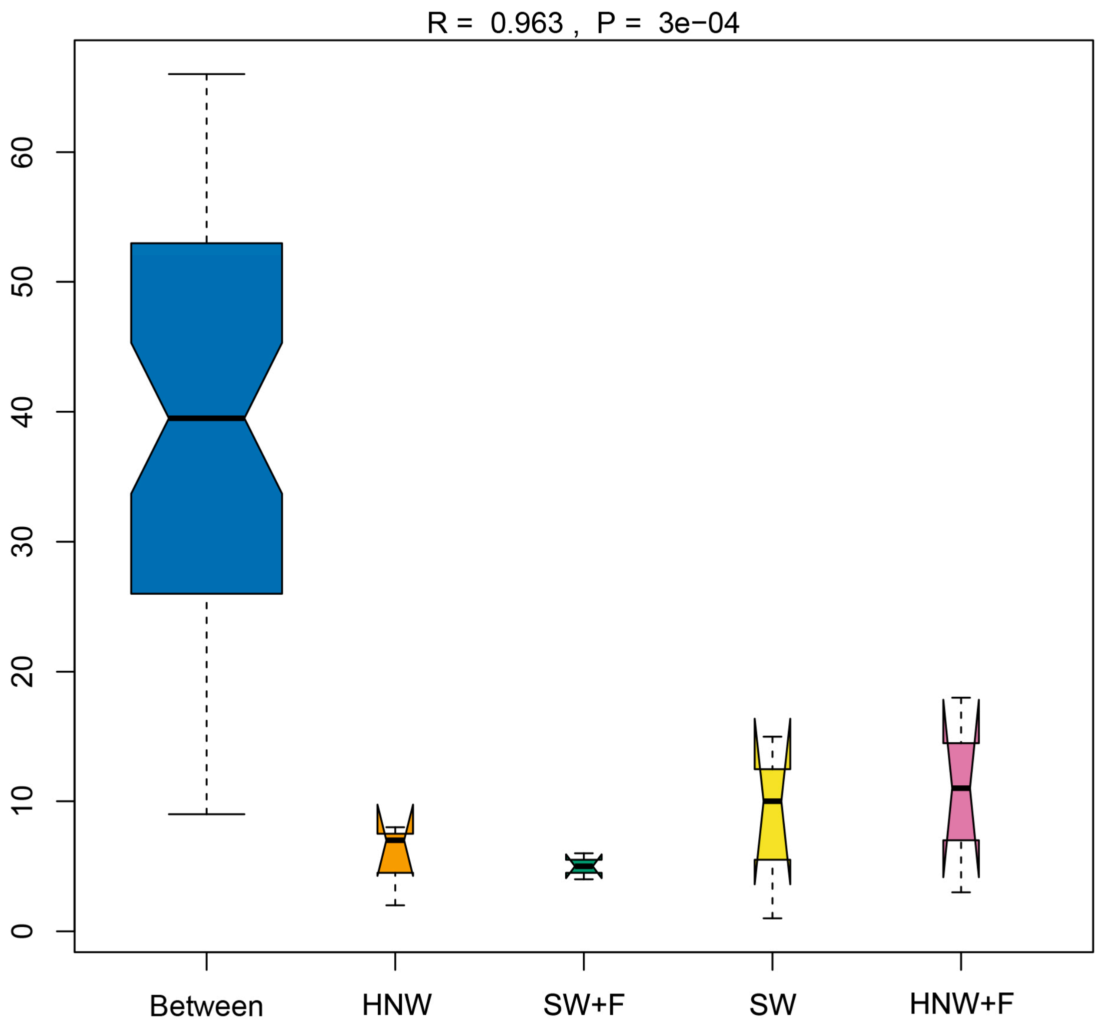

Figure S3 Analysis of similarities (ANOSIM) based on Bray-Curtis dissimilarity at the genus level.

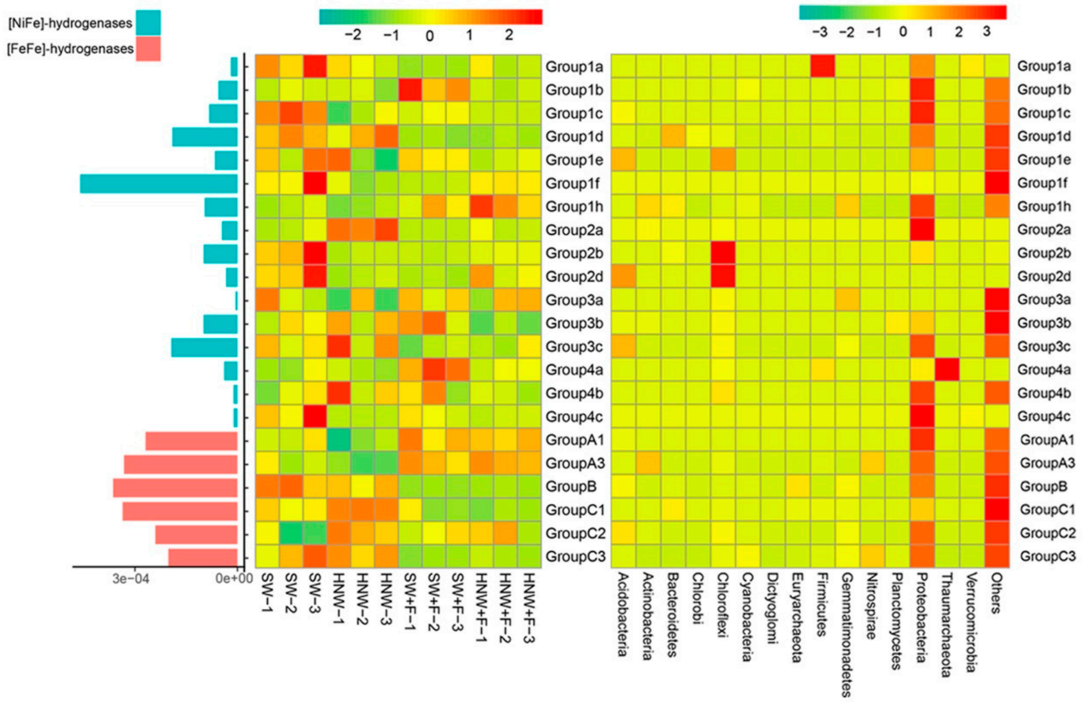

Figure S4 Relative abundances of rhizospheric hydrogenase genes. Bars indicate the total relative abundances of hydrogenase genes across all treatments. The middle heatmap represents the relative abundances in each sample, and the right represents the relative abundances in each dominant phylum.

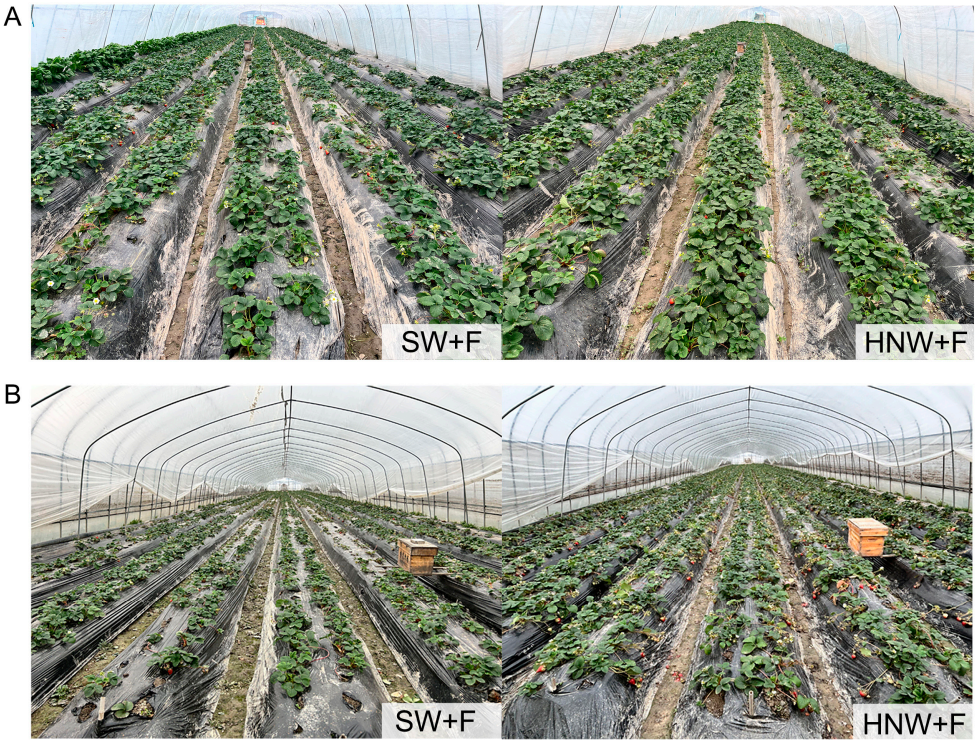

Figure S5 Photographs of strawberry field and representative plants were taken on January 14, 2022 (A) and February 14, 2023 (B).

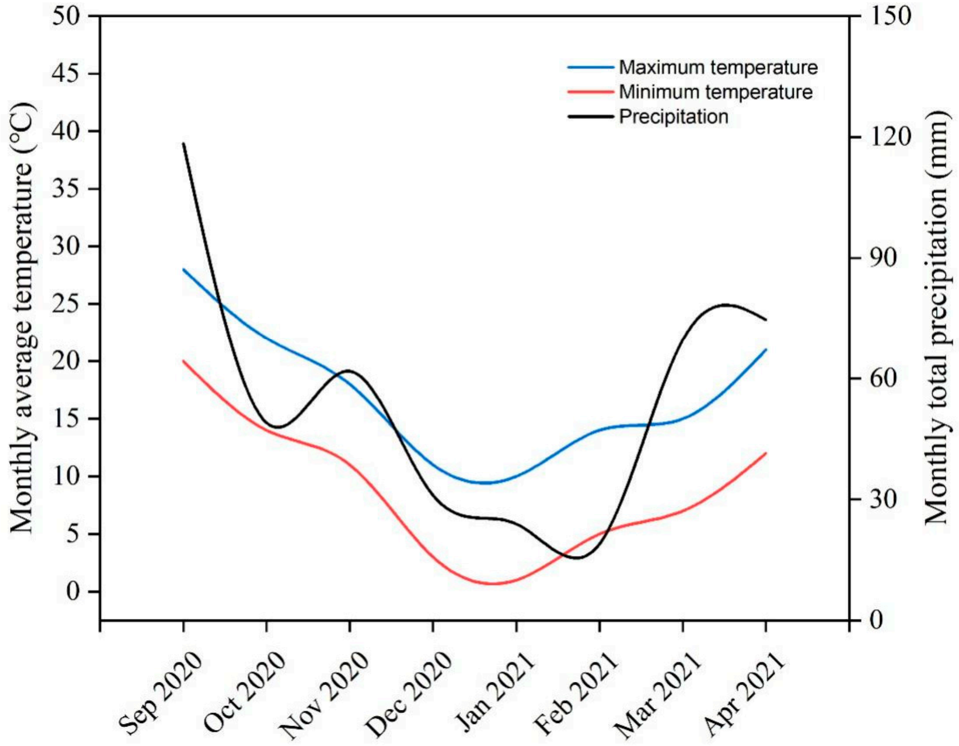

Figure S6 The monthly maximum and minimum temperature and total precipitation during strawberry planting.
